# Damage Detection with Streamlined Structural Health Monitoring Data

**DOI:** 10.3390/s150408832

**Published:** 2015-04-15

**Authors:** Jian Li, Jun Deng, Weizhi Xie

**Affiliations:** 1 Guangdong Provincial Academy of Building Research, Guangzhou 510500, China; E-Mail: jil2sun@126.com; 2 School of Civil and Transportation Engineering, Guangdong University of Technology, Guangzhou 510006, China; E-Mail: wzxie@outlook.com

**Keywords:** damage detection, data compression, data communication, knowledge discovery, sensor network, structural health monitoring, system identification

## Abstract

The huge amounts of sensor data generated by large scale sensor networks in on-line structural health monitoring (SHM) systems often overwhelms the systems’ capacity for data transmission and analysis. This paper presents a new concept for an integrated SHM system in which a streamlined data flow is used as a unifying thread to integrate the individual components of on-line SHM systems. Such an integrated SHM system has a few desirable functionalities including embedded sensor data compression, interactive sensor data retrieval, and structural knowledge discovery, which aim to enhance the reliability, efficiency, and robustness of on-line SHM systems. Adoption of this new concept will enable the design of an on-line SHM system with more uniform data generation and data handling capacity for its subsystems. To examine this concept in the context of vibration-based SHM systems, real sensor data from an on-line SHM system comprising a scaled steel bridge structure and an on-line data acquisition system with remote data access was used in this study. Vibration test results clearly demonstrated the prominent performance characteristics of the proposed integrated SHM system including rapid data access, interactive data retrieval and knowledge discovery of structural conditions on a global level.

## 1. Introduction

The importance of structural health monitoring (SHM) in the design, construction, maintenance and post-extreme event repair of civil engineering structures has been recognized in the past decade. Vibration-based SHM methods have the potential to detect damages in structures in a global sense [1,2,3,4]. Many damage detection methods that examine changes in the vibration characteristics of monitored structures have been devised although quantitative assessment of structural damages using real vibration data collected from civil engineering structures still remains to be a challenging task today.

Structural health monitoring can be considered in a statistical framework. Bayesian methods have been extensively applied to system identification [5] and structural health monitoring [6,7]. The detection and location of damage can be formulated as a hypothesis test, with the location often determined by modal parameter estimation [8,9]. The widely studied structural system identification method could be seen as a feature extraction method specially fitting the SHM applications [10]. The system identification methods, including frequency-domain methods [11,12,13] and time-domain methods [14], extract system properties such as modal frequencies, modal shapes, and stiffness from a set of vibration measurements.

**Figure 1:**
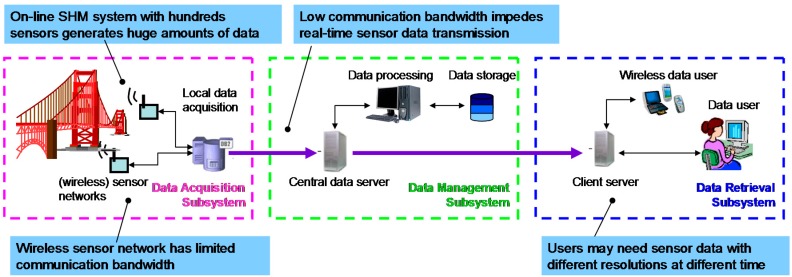
Schematics of an on-line structural health monitoring system and technical challenges.

On-line SHM enables automatic collection of vital information about civil infrastructures’ operating condition and in the aftermath of a catastrophic event such as a strong earthquake, its ability to continue to carry the design load. There is a strong and growing interest in on-line SHM systems among engineers, researchers, and decision makers in civil engineering applications. An on-line SHM system is generally comprised of three subsystems—data acquisition subsystem, data management subsystem and data retrieval subsystem. Figure 1 shows the schematics of such an SHM system with wireless communication and emerging technological challenges in its subsystems. Compared with damage detection methods, issues related to sensor data transmission and data management in on-line SHM system have been studied to a lesser extent. It is seen that one of the issues of emerging importance to current and future structural health monitoring practice is to streamline and speed up the data flow in on-line SHM systems.

For on-line SHM system, sensor data need to be transferred from the local site to a central data processing station, and accessed by emergency workers, engineers, and decision makers. Due to its large scale and complexity, structural condition monitoring of civil engineering structures generally requires a substantial number of sensors of different types. The huge amounts of sensor data generated by the large scale sensor networks of on-line SHM systems often overwhelm the systems’ capacity for data transmission and analysis; this inconsistency in the data handling capacity of the different components of on-line SHM system has impeded its effective use. This is especially true during and immediately after an extreme event when communication bandwidth may become very limited due to infrastructure damage and an increase in emergency communication use. Special consideration of its data flow aspect when designing and implementing an on-line SHM system will be very beneficial to enhancing its overall performance such as streamlined data flow and reduced load for data analysis. This is especially true for systems involving wireless sensor networks or wireless communication, which typically have a limited communication bandwidth. In addition, the emphasis on data flow by using high-performance information processing algorithms will be beneficial to alleviate the data transmission and power consumption problems in SHM systems with wireless sensor networks.

This paper presents a new concept for an integrated SHM system, in which the data flow in the SHM system is streamlined and is used as a unifying thread to integrate the individual subsystems of the SHM system. Prominent performance characteristics of such an integrated SHM system include: sensor data size is reduced before transmission by specially designed sensor data compression methods [15]; an interactive data retrieval method further alleviates the demand on communication channels by transmitting only selected data set and provides data users with the flexibility of selecting data resolution and time windows to view and download data through feature monitoring [16]; structural knowledge discovery is based on a second order structural system identification method [17]. To examine this concept in the context of a vibration-based SHM system, real sensor data from an on-line SHM system comprising a scaled steel bridge and an on-line data acquisition system with remote data access was used in this study.

## 2. Concept for Integrated SHM System

Aimed toward enhancing the system’s efficiency and robustness by streamlining data flow, the concept for an integrated SHM system, which is founded on recent technology advances in sensor networks, wireless communication, and information technology, is briefly described in this section.

In the design of our integrated SHM system, emphasis is placed on the data flow aspect of the system, in which streamlined data flow is viewed as a thread to integrate the individual components of the on-line SHM system. In this way, the data generation, data handling and analysis capacities of different parts of an on-line SHM system can be made more uniform than in current practice. For example, sensor data size is reduced before transmission to reduce the time latency caused by data transmission; an interactive data retrieval method is applied to provide the data user the flexibility of selecting which data set and what data resolutions to download; to assist with decision making, a second order structural system identification method is used for structural knowledge discovery (e.g., condition assessment, *etc.*).

The integrated SHM system involves the use of a variety of recently developed information processing algorithms [15]. More specifically, the integrated SHM system has the following prominent functionalities:

(1) Efficient Data Transmission: High-performance sensor data compression methods that are custom-designed for large scale on-line SHM system can alleviate the data transmission problem associated with the limits bandwidth of communication networks, as shown in Figure 1. Sensor data compression also provides a promising approach to enabling rapid data access by engineers and decision makers immediately after extreme events. A family of linear predictor-based lossless sensor data compression methods [18] will be implemented in the data acquisition subsystem of an integrated SHM system: the linear predictor-based lossless data compression method for single sensor is to be embedded on a wireless sensor board to accelerate data transmission; in master nodes of wireless sensor network and local data acquisition stations, where sensor network data becomes available, the linear predictor-based compression method for sensor network data will be implemented to reduce the size of sensor network data.
(2) Interactive Data Retrieval: As mentioned earlier, a SHM system with a large scale sensor network generates a huge amount of sensor data. This huge data size would also increase the burden on data analysis, which may be necessary only for selected sensor data. A principal component analysis (PCA)-based interactive data retrieval method will be applied to the data management subsystem of the integrated SHM system to enable interactive retrieval of sensor data. In doing so, data users can decide whether to download data by first observing the pre-defined features. If data downloading is necessary as determined by the data users from the monitored features, the data users will have the flexibility to interactively retrieve the sensor data by selecting which data window and what data resolution to retrieve. Before sending them to communication channels for transmission, the selected data will be compressed using a method which involves the above-described linear predictor-based data compression method and PCA transform.
(3) Structural Knowledge Discovery: After receiving the progressively downloaded data with desired resolution, a structural knowledge discovery method will be applied for reliable condition assessment. In this study, a second order structural parameter identification method [17], which can extract structural stiffness coefficients from output-only measurements, will be used for this purpose. Damage detection based on second order structural parameter identification is executed only when sensor data deserves a close examination as decided by feature monitoring. Users can use other structural knowledge discovery methods which can reliably detect the occurrence of potential damage during extreme events as well as to locate and possibly quantify the damage, and predict the remaining structural capacity. This functionality of the integrated SHM system will be implemented in its data retrieval subsystem (see Figure 1).

Consideration of streamlined data flow in the design and implementation of on-line SHM systems is particularly important to systems with wireless sensor networks or wireless communications. Wireless sensors offers several benefits over conventional wired sensors and recently have received considerable interest among civil engineering researchers [19,20,21,22]. Wireless sensors are easy to deploy, and possess flexibility in sensor network configuration. A wireless sensor network may consist of hundreds of wireless sensor nodes spread over a civil infrastructure system. A multi-hop wireless sensor network is typically used to relay sensor data from remote wireless sensor nodes to a base station through several routers. Limited communication throughput and short battery life are currently two bottlenecks that impede the widespread use of wireless sensor networks in on-line SHM systems. Therefore, the concept of integrated SHM system with an emphasis on data flow by using high-performance information processing algorithms has a potential to alleviate the data transmission and power consumption problems in SHM systems with wireless sensor network.

## 3. Component Technologies in the Integrated SHM System

The technologies proposed for the various components of an integrated SHM system which include sensor data compression, interactive data retrieval, structural knowledge discovery through structural system identification and statistical control chart analysis, are briefly reviewed in this section.

### 3.1. Lossless Sensor Data Compression

The linear predictor-based lossless data compression methods are a two-stage procedure developed for compressing vibration sensor data [18]. In the first stage, the original data sequences are transformed into predictor residue sequences by linear predictors derived in a system identification framework. The predictor residues generally have smaller amplitude range and more concentrated distribution that yield smaller information entropy. Information entropy is defined as a measure of information content in messages by Shannon [23]. The second stage involves entropy coding in which arithmetic coding is used for binary codeword assignment which represents the predictor residue sequence. The binary code sequence is the final compressed data to be transmitted. After receiving the transmitted data, a data reconstruction procedure, which is the inverse of above-described two-stage procedure for data compression, will be employed to recover the original sensor data.

For different components of an integrated SHM system, different predictor models may be applied for sensor data compression. In wireless sensor nodes where only data from the sensor itself is available, the linear predictor-based sensor data compression method for single sensor data will be used. In the local data acquisition station or cluster head of wireless sensor network where multiple sensor data is available, the linear predictor-based sensor data compression algorithm for sensor network will be implemented to achieve enhanced compression performance through the use of an AR model-based linear predictor [18]. The AR model-based predictor can model the spatial correlation existing between measured data from different sensors distributed over the same structure, which is not considered in the linear predictor for single sensor. Details of predictor design and identification of predictor parameters can be found in Reference [18]. The linear predictor-based sensor data compression methods are lossless methods which do not cause any signal distortions.

### 3.2. Interactive Sensor Network Data Retrieval

In order to facilitate efficient data transmission and remote data retrieval, an interactive sensor data retrieval method based on principal components analysis (PCA) transform is developed [16], which has the following desirable features: (i) the occurrence of major events including abnormal excitation and structural damages can be monitored before transmitting original sensor data; (ii) data corresponding to major events can be retrieved progressively with coarse-to-fine resolution levels according to users’ requirements; (iii) data size is reduced using the PCA transform and linear predictor-based data compression method.

The PCA transform-based interactive data retrieval method has two major functionalities including feature monitoring and progressive data transmission. For an on-line SHM system requiring rapid data access, downloading all available data would impose a heavy communication bandwidth and data analysis burden. Therefore, acquiring qualitative knowledge about system conditions to assist decision making about which data set to download is highly desirable before downloading and analyzing large amounts of sensor data. In the PCA transform-based method [16], eigenvalues of the covariance matrix of the sensor network data are used for feature definition. Changes in the monitored feature might indicate the changes in input excitation, structural properties, noise level, or other interested events for data users. The purpose of combining eigenvalues is to condense as much as possible information from multiple eigenvalues into a single feature. The PCA based feature which has a much smaller data size than the original sensor data is first transmitted to data users. Data retrieval and detailed data analysis will be performed only when the PCA feature suggestions it is necessary.

The other functionality of the interactive data retrieval method is progressive data transmission, which provides users the flexibility of selecting data resolutions based on their particular need. One of the most important properties of PCA transform is that any number of principal components can be used to reconstruct the original data at a price of information loss. The first few principal components carry the most important information of the original data. Therefore, instead of using all the principal components to reconstruct data, we may represent the data in terms of the first few principal components. Transmitting and reconstructing only the first few principal components can preserve the basic information of the original sensor data with a reasonable level of fidelity. Data users at remote sites can determine whether the sensor data being requested is worth further examination by first taking a look at the general waveform of the coarser data, and in doing so users can save time and transmission cost in retrieving sensor data.

Additionally, since the principal components generated from PCA transform are uncorrelated to each other, PCA transform is also beneficial to data compression by decomposing spatial correlations in original sensor data. In this interactive data retrieval method, data compression is performed before sending the data to communication channels. The data compression process concerned involves the use of PCA transform, linear predictor and arithmetic coding to remove the spatial, temporal correlation and redundancy associated with binary codeword assignment respectively. The original data can be reconstructed from the compressed data through an inverse three-step procedure [16].

### 3.3. Knowledge Discovery through System Identification and Statistical Analysis

#### 3.3.1. Second Order Structural Parameter Identification

In the integrated SHM system, a time-domain structural system identification method is used for structural knowledge discovery. This method can directly identify second order structural parameters such as stiffness, mass, and damping ratios directly from vibration sensor data.

This structural system identification method starts with expanding the state space model into linear models which have parameters as functions of second order structural parameters. Prediction Error Minimization (PEM) method is then used to estimate the unknown second order structural parameters. The final mathematic models to be identified are: 

If the input excitation is available:

(1) $$\sum_{i-0}^{2N}\alpha_{i}(\theta )\times y_{k-i}=\sum_{i=0}^{2N}\beta_{i}(\theta )\times u_{k-i}+\sum_{i=o}^{2N}\gamma_{i}(\theta )\times e_{k-i}$$

where $u_{k}\in {ℜ}^{N}$ is the system input, $y_{k}\in {ℜ}^{m}$ is the system output due to input $u_{k}$; $e_{k}\in {ℜ}^{m}$ is called the innovation and is assumed to be a zero-mean Gaussian white system noise. $\alpha_{i},\beta_{i},\gamma_{i}$ are system coefficients related to the system parameter vector θ. *N* is degrees-of-freedom (DOF).

For output-only measurement:

(2) $$\sum_{i-0}^{2N}\alpha_{i}(\theta )\times y_{k-i}=\sum_{i=o}^{2N}\gamma_{i}(\theta )\times e_{k-i}$$

The unknown second order structural parameters to be identified are denoted as vector θ. To identify the model described in Equation (1), both excitation force and structural response measurements are needed. The whole set of second order structural second order parameters including mass, stiffness and damping ratios can be identified simultaneously. The model described in Equation (2) is based on the Gaussian distributed ambient excitation assumption. Only response measurements are necessary to identify this model. Due to lack of information about excitation, only part of second order parameters (*i.e.*, either mass or stiffness) can be identified from this model.

In this method, the structure is assumed to be a *N*-degree of freedom system*.* The linear model coefficients $\alpha_{i},\gamma_{i}$ are M by M matrices, $\beta_{i}$ is M by L matrix, L is the number of excitation measurements sensors and M is the number of response measurements sensors. Mathematically, only if the number of unknown parameters $\alpha_{i},\beta_{i},\gamma_{i}$ is bigger than physical parameter θ, it is always possible to get θ from this methods by PEM method. But, with fewer sensors and fewer actuators, less $\alpha_{i},\beta_{i},\gamma_{i}$ can be determined and $\alpha_{i},\beta_{i},\gamma_{i}$ are easier to be affected by local sensor noise. Meanwhile, $\alpha_{i},\beta_{i},\gamma_{i}$ might be only correlated to part of θ. So, less accurate θ can be determined. More information about this method can be found in reference [17].

Based on these two models, a two-stage procedure for structural health monitoring has been developed [17]. In this two-stage SHM method, the first stage entails the identification of all second order structural parameters of the original structure from well-controlled vibration tests with known input. The second stage involves output-only structural system identification which is targeted for ambient vibration applications with unknown inputs. In the second stage, the structural masses identified from Stage one is assumed not to change and only stiffness parameters will be identified from output only measurements. Damage is located and quantified through the changes in the identified stiffness coefficients.

Since this system identification method can directly extract second order structural parameters which are mathematical descriptions of structural physical properties, the identification results provide structural knowledge regarding damage locations, damage severity and possibly remaining capacity of the structure. Details of this system identification method can be found in References [15,17].

#### 3.3.2. Statistical Control Chart Analysis of Feature and Identified Stiffness

The PCA transform and system identification can only be applied to a data set with limited time duration. Therefore, each data set may have local properties because of its limited time duration. Features such as the aforementioned PCA feature and identified stiffness parameters extracted from different data set might fluctuate within a particular range of values even though they are from the same structure. It is thus necessary to define the confident range of the extracted features which can be used to classify the structural system into conditions with different levels of damage. A statistical control chart analysis is performed here to analyze these features from a statistical point of view. To plot the statistical control chart, the confident range of features must be calculated from historical data. To define the feature range with α% confidence, the upper and lower control limits (denoted as UCL and LCL respectively) can be expressed as [24]:

(3) $$UCL=invnorm(1-(\alpha /2)\%,\overset{-}{F},S_{F})$$

(4) $$LCL=invnorm((\alpha /2)\%,\overset{-}{F},S_{F})$$

where invnorm( ) denotes the inverse function of the probability density function of normal distribution; $\overset{-}{F}$ and $S_{F}$ are the mean value and standard deviation of each feature including the PCA feature and identified stiffness coefficients as defined in this study. Equations (3) and (4) are based on the normal distribution assumption for the features extracted from historical data. However, it has been shown that the control limits based on the normal distribution assumption can often be satisfactorily used unless the population is extremely non-normal [24,25]. α% is the confidence value that is used for system classification. Overly large values of α% will lead to a higher chance for misclassifying systems with change. Since structural damages are always reflected as stiffness reduction in this study, only the lower control limit for identified stiffness coefficients is considered for damage quantification.

## 4. Vibration Tests on Scaled Steel Bridge Structure

### 4.1. Steel Bridge Structure and Monitoring Procedure

**Figure 2:**
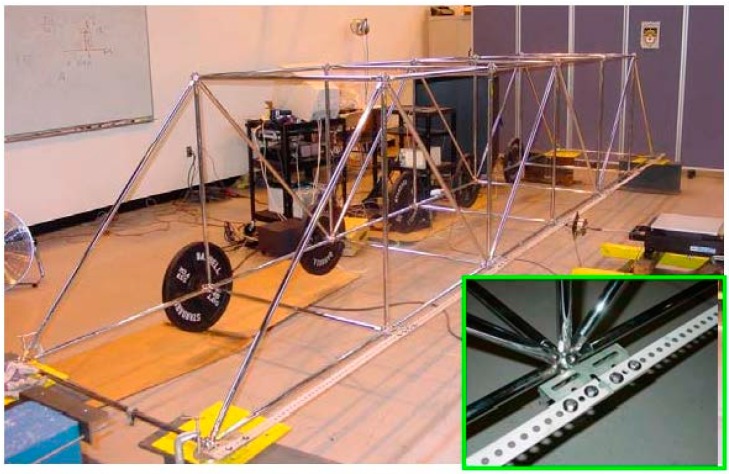
View of scaled steel bridge structure (inset shows the connection detail).

To demonstrate the concept of integrated SHM system, a vibration-based SHM system with remote data access was set up to monitor a scaled steel bridge structure, as shown in Figure 2. This steel bridge structure measures 1-m × 1-m × 6-m (for the width, height and length, respectively). The steel bridge structure has a total number of 24 nodes, 32 short members with a 1-m length and 12 long members with a 1.414-m length, made by the MeroForm Systems [26]. The four end nodes in the lower plane of the steel bridge structure are restrained for translational motion at the bridge’s supports. Five 45-lb (20.4 kg) steel weights are each attached to the lower nodes along one side of the lower plane to simulate structural mass. Additional slotted steel stripes are bolted to all seven nodes along the opposite side of the lower plane to provide additional stiffness in the transverse direction of the steel bridge structure. A close-up view of the stripe connection is shown in the inset of Figure 2. By loosening the connections of these steel stripes, structural damages can be physically simulated in vibration tests. The first five modal frequencies of this steel bridge structures are: 1.64, 3.96, 6.24, 9.00, and 11.09 Hz.

Five accelerometers (Model 393B04, PCB Piezotronics Inc., Buffalo, NY, USA) were used to measure the vibration responses of the steel bridge structure. The accelerometers were attached to the nodes along one side of the lower plane of the steel bridge structure. A real-time data acquisition system (Model RTMS-2001, Digitexx Data Systems Inc., Pasadena, CA, USA) was used for recording and broadcasting sensor data to Internet. Using real vibration data collected from this prototype system, factors that could influence the performance of integrated SHM system could be examined.

A time window is adopted to divide the continuously measured data into individual data sets. Each measurement set is denoted as one test in this study. One test thus defined includes 1024 data samples from each accelerometer and therefore the entire data set contains a total of 5120 data samples. Since the sampling rate used by the data acquisition system was 200 Hz, the time window for dividing the measurements was therefore equal to 5.12 s. By adopting this time window to perform operations on each data packet, the overall decision delay in the entire SHM system is only 5.12 s if the computation delay is neglected. The test procedure for the steel bridge structure is divided into the following three steps:

(1) Step 1: Well-controlled vibration test to identify the second order structural parameters of the steel bridge structure.
(2) Step 2: Output-only vibration test on the original (undamaged) bridge structure to identify its initial parameters.
(3) Step 3: Vibration tests which simulate various damage scenarios to demonstrate the concept of the integrated SHM system.

### 4.2. Pre-Tests for Identification of Second Order Structural Parameters

Initial parameters of the steel bridge structure, which serve as a baseline for future analysis, need to be identified first using test data. The parameters to be determined from pre-test measurements include:

(1) Predictor parameters: the predictor parameters for the linear predictor-based sensor data compression methods that need to be determined from pre-test data include the predictor order and predictor coefficients.
(2) Statistical control limits of PCA-based features: these parameters will be used to build a control chart in the statistical control chart analysis.
(3) Mass and damping parameters of the steel bridge structure: a well-controlled vibration test needs to be conducted to identify the system’s mass and damping ratios in the first stage of the vibration-based SHM procedure [17].
(4) Control limits of identified stiffness parameters: control limits of the identified stiffness that will be used to enhance the accuracy of damage detection in later on-line monitoring stage need to be established from a statistical analysis of pre-test data.

**Figure 3:**
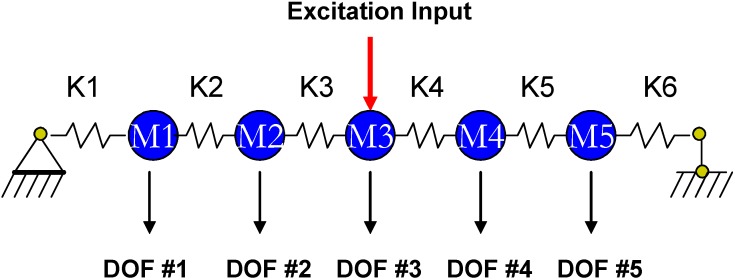
Idealized lumped-mass model for the steel bridge structure.

For output-only monitoring of its structural stiffness parameters, the mass and damping ratios of the steel bridge structure need to be identified beforehand with a well-controlled vibration test (*i.e.*, Step 1 test as described in the preceding section). A well-controlled vibration test is defined as: (i) measurements of the input excitation are available and must be force measurements; (ii) the co-location requirement needs to be satisfied, where co-location means that there exists at least one degree-of-freedom (DOF) with both a sensor and an actuator. To identify the second order structural parameters, the original steel bridge structure is idealized into a 5-DOF lumped-mass model as shown in Figure 3. A total of 16 unknown parameters thus need to be identified as second order structural parameters including five masses, five damping ratios and six stiffness parameters.

In the well-controlled vibration test of this study, a long stroke shaker with a 100-lb maximum output force was used to excite the steel bridge structure at its middle node located in the lower plane and the input dynamic force was measured using a load cell (Sensotec Model 45, Honeywell Inc., Morristown, NJ, USA). A total of six identical tests were conducted with 1024 data samples from each sensor. The measured data set was used to identify the second order structural parameters of the bridge structure using the method described in [17]. The average values of the mass and damping ratios from these six tests were taken as the parameters used in the subsequent on-line monitoring stage. All 16 unknown parameters can be identified simultaneously from the measurements in each test.

In Step 2, a total of 151 vibration tests each with a duration of 5.12 s were conducted on the original bridge structure to identify the above-described parameters for future monitoring use. In all the vibration tests conducted in Step 2 (see Section 4.1), acceleration responses of the bridge structure were the only quantities to be measured. Optimally selected parameters identified from a large ensemble of vibration data can capture the statistical properties of the original bridge structure and hence these parameters will be used for future tests on the same structure with much change in the system. In practice, these parameters can be periodically updated using most recent measurements from ambient vibration test.

**Figure 4:**
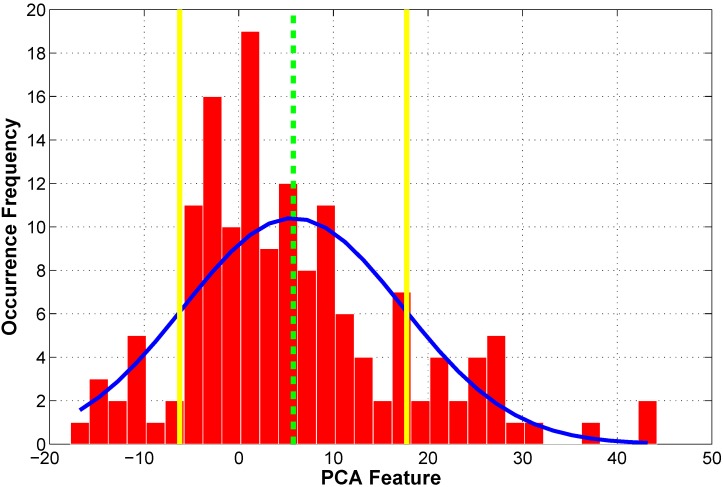
Histogram of PCA feature and its control limits.

Figure 4 plots the histogram of the PCA feature calculated using data from the 151 pre-tests. Also shown in Figure 4 is a normal distribution curve with the same mean and variance values as the PCA feature. The dash line in Figure 4 indicates the mean value of the PCA feature. The solid lines denote the UCL and LCL values for the PCA feature when α is equal to 70 in Equations (3) and (4). An interval with 70% confidence was adopted because a strict condition is preferred to define the confident interval of the original system. Data downloading is considered necessary if the extracted PCA feature falls within this range.

**Figure 5:**
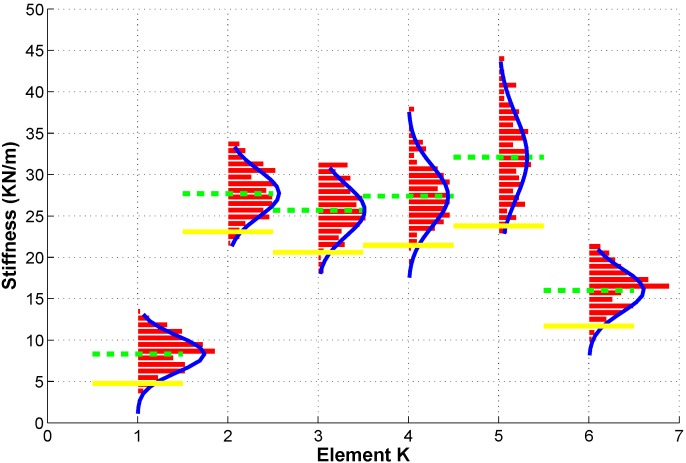
Control chart of identified stiffness coefficients.

The variation of the stiffness coefficients identified from each test data set was also observed in the pre-test identification results. The control chart analysis was therefore used for the identified stiffness coefficients. The objective of setting the control limits for the identified stiffness parameters is to confidently classify damaged structures from undamaged structures based on the identified stiffness values. An identified stiffness value which falls within the confident range of the undamaged structure implies that the likelihood for the structure to be damaged is low. Since structural damage in this study is characterized as stiffness reduction, only lower control limit needs to be established here. Another approach to analyzing damage based on identified stiffness control chart is to compare the mean values of identified stiffness parameters from several repetitive tests with the corresponding stiffness values from pre-test data. Even if a single identified value cannot be confidently classified as being damaged using the control limits because of the variation, the confidence of classification can be enhanced by comparing mean values. Therefore, constructing the control limits for identified stiffness coefficients include the calculation of lower control limit and mean values for the identified stiffness. Figure 5 shows a control chart which can be used for damage detection. Mean values and lower control limit for the member stiffness of the lumped-mass model of the steel bridge structures are also shown in Figure 5 as dash line and solid line respectively. Normal distribution curves for different member stiffness are also plotted in Figure 5.

### 4.3. Performance of Integrated Structural Health Monitoring

After identifying the initial parameters from pre-test data, the vibration-based SHM system is ready for on-line structural health monitoring use. In this experimental study, three damage scenarios were simulated in the steel bridge structure to demonstrate the concept of integrated SHM system. These three damage scenarios, which correspond to structure with damage at single location, structure with damage at multiple locations, and retrofitted structure respectively, are briefly described below:

(1) Damage scenario #1: the steel stripe connection at K1 location was completely separated from the primary structure by loosening the bolts.
(2) Damage scenario #2: the steel stripes at K3, K4 and K5 locations were simultaneously separated from the primary structure to simulate the multiple location damage case.
(3) Damage scenario #3: all steel stripes were connected back to the primary structure by tightening the connection bolts to simulate the retrofitted structure case.

Stiffness reduction is introduced to the particular locations where the stripe connection was disattached from the primary structure. Due to the configuration of the steel bridge structure, loosening the steel stripe connection at one location would also influence the stiffness of other spans, particularly those in neighboring spans. For each damage scenario test, a total of 15,360 data samples were continuously measured from each of the five accelerometers. These measurements is then divided into 15 tests (15,360 = 15 × 1024) using a predefined time window. The sampling rate was 100 Hz. From here forth, the results will be presented in terms of test numbers instead of data sample numbers. The input excitations in all these damage scenarios were Gaussian white noise to simulate ambient excitation in real structures.

#### 4.3.1. Damage Scenario #1

**Figure 6:**
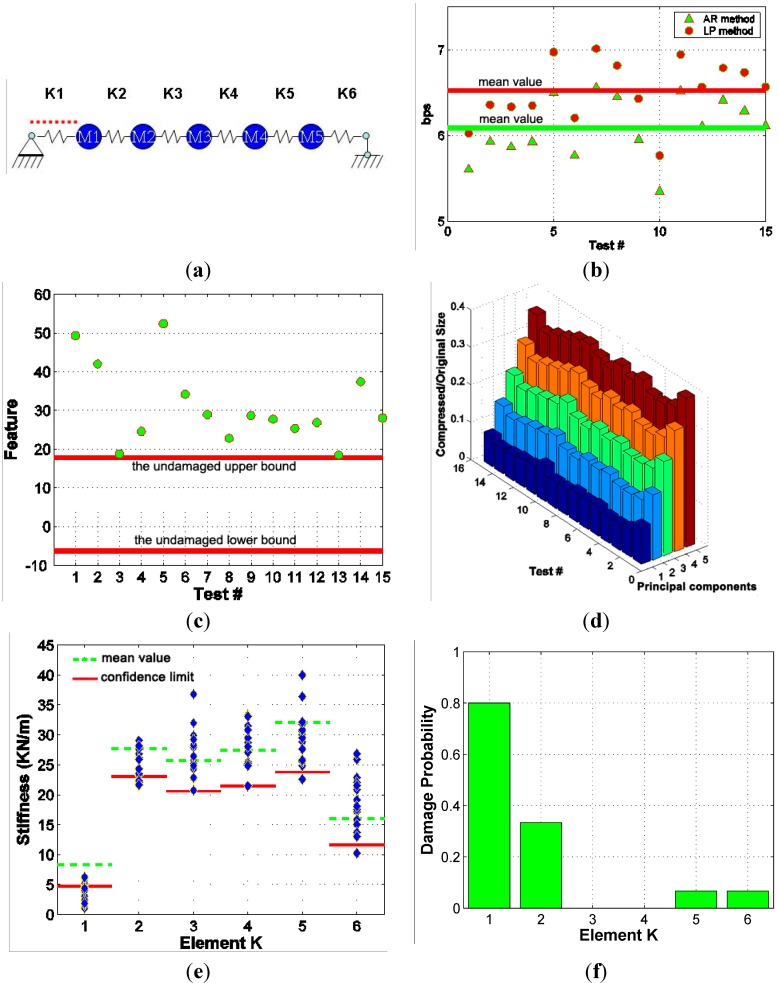
Performance of the Integrated SHM system in Scenario #1 (**a**) Damage at single location; (**b**) Sensor data compression; (**c**) Feature monitoring; (**d**) Progressive data transmission; (**e**) Identified stiffness; (**f**) Statistical analysis of damage.

Figure 6 plots the results of integrated structural health monitoring of the steel bridge structure in damage scenario #1. At the local data relay or central data processing station, the linear predictor-based sensor data compression method for sensor network data (denoted as the AR method) is applied. By considering the spatial correlation between different sensor data, the AR method can achieve higher compression ratio than the linear predictor-based sensor data compression method for single sensor (denoted as the LP method). Figure 6b shows the compression results of the LP method and the AR method. The bit rate shown here is the average bit rate of all five sensor data sequence in each test. The solid lines in Figure 6b indicate the mean values of the average bit rates. 

It is seen that the AR method can further reduce the bits rate of sensor data by about 0.6 bits per sample compared to the LP method. This reduction in bit rates will translate into a higher compression ratio which will further reduce the time consumption required for sensor data transmission. When sensor data is received at the data management subsystem, the interactive data retrieval method which is based on PCA transform will be applied. This PCA-based method integrates feature monitoring, progressive data transmission, and near-lossless data compression. Using the interactive data retrieval method, raw sensor data are preprocessed and stored as principal components on the data server in the data management subsystem. Pre-defined PCA features are first transmitted to data users to assist with data retrieval decision before downloading the actual sensor data. Monitoring this feature can help data users make decisions on which data to retrieve at what resolution levels. As shown in Figure 6c, the solid lines represent the upper and lower bounds of the PCA feature corresponding to the original (undamaged) structure in the pre-test. If the feature goes outside the region defined between these two limits, it indicates the possible occurrence of major events such as changes in system properties, changes in excitation input (e.g., amplitude increase) or even changes in sensor itself (e.g., bad sensor). The results shown in Figure 6c clearly show that the features calculated from the 15 test data corresponding to damage scenario #1 fell outside this region although it did not tell what type of changes have occurred in the system. Major changes in the feature being monitored certainly warrant the need for downloading the data with increased resolution levels.

Since each principal component contains different amount of information, the PCA-based interactive data retrieval method would allow data users to download sensor network data progressively starting with the most important principal components. These principal components will also be compressed by the LP-based compression method before transmission. By doing so, data can be downloaded and reconstructed progressively in a coarse to fine manner (*i.e.*, with increased resolution). Therefore, before downloading all principal components is completed, only the key information is received by the data user. To measure the signal distortion induced by transmitting principal components progressively, signal-to-noise ratio (SNR) defined in Equation (5) is used:

(5) $$\text{SNR}=20\log_{10}\left(\sum_{i}\left|x_{i}\right|/\sum_{i}\left|\Delta x_{i}\right|\right)$$

where *x_i_* is the original data at *i*-th time step and Δ*x_i_* is the difference between original data and reconstructed data at *i*-th time step.

Figure 6d shows the gradually increased data size and corresponding increase of SNR values when progressively downloading the principle components. If all five principle components (because a total of five accelerometers were used in this study) were downloaded, the data size would be about 35% of the uncompressed data size with an SNR value of 60, which implies minimal signal distortion to the transmitted data.

Finally, the downloaded data corresponding to the major events (as determined from the observed feature changes) will be analyzed using the second-order structural system identification method. This system identification method directly identifies structural stiffness from ambient vibration data without the need for excitation input measurement. The mass and damping ratio identified from well controlled vibration test were used for the stiffness identification. The statistical analysis of the identified stiffness during the pre-test stage provides the confidence limits for the structural stiffness. An identified stiffness value lower than the confidence limit will be classified as damage with 90% confidence. A comparison of the mean values of the identified stiffness of the original structure (presented as the dash line) and those of damaged structures in Figure 6e provides an alternative way of damage detection. Figure 6e shows the identified stiffness for the six spans of the steel bridge structure from all 15 tests. Figure 6f shows the damage probability of the individual member stiffness. It is seen in Figure 6e that member stiffness K1 was outside the limits 12 times (out of a total 15) and the mean value of these 15 identified stiffness values was much smaller than the corresponding mean value for the original structure. Therefore, we can confidently conclude that the K1 was damaged. For K2, although only five identified results from these 15 tests are confidently classified as being damaged, other identified values within the limits also show certain level of reduction compared with the mean value of the pre-test results. Certain occurrence frequencies of confidently classified as being damaged and the apparent difference between the average stiffness value of these 15 tests and that of the 151 pre-tests indicate that K2 had some smaller level of damage. The reduction of the mean values of identified stiffness shows that the level of damage in K2 was less severe than that in K1. Other stiffness parameters such as K4 cannot be classified as being damaged since no or only one identified result out of a total of 15 identified stiffness values went outside the pre-defined limits and yet no significant change was observed in average stiffness values. Therefore, the structural knowledge finally acquired is that K1 was severely damaged and K2 was slightly damaged, as also shown in Figure 6f. Since the identified feature was the structural stiffness, the remaining capacity of the damaged structure may be predicted based on these identified results.

#### 4.3.2. Retrofitted Scenario #2

**Figure 7:**
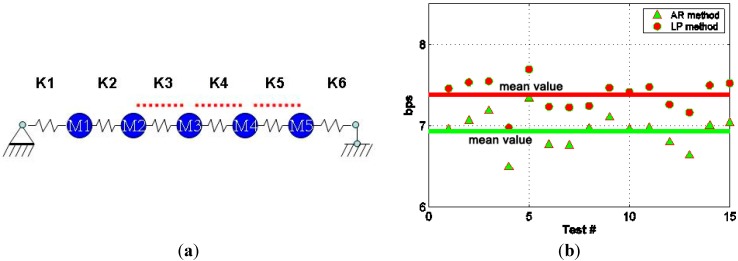

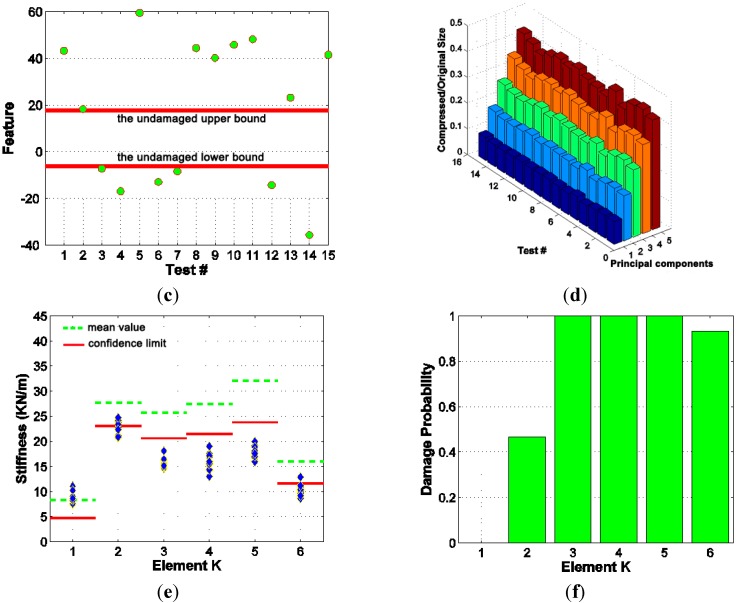
Performance of Integrated SHM system in Scenario #2 (**a**) Damage at multiple locations; (**b**) Sensor data compression; (**c**) Feature monitoring; (**d**) Progressive data transmission; (**e**) Identified stiffness; (**f**) Statistical analysis of damage.

This case is presented to examine the performance of the proposed integrated SHM system under multiple damage situations. The steel stripes at K3, K4 and K5 locations were all disconnected from the primary structure by completely loosening the connections in this case. The difference between this scenario and other cases was first noted in the performance of the various data compression methods. The bit rate of the compressed data by both the LP and AR methods was increased compared to the results as shown in Figure 7b. 

The reason for this phenomenon is that increased damage level worsened the performance of linear predictors identified from the pre-test data. Another notable change is on the features defined for the PCA-based interactive data retrieval method. This feature simply shifted away from the pre-defined confident range in damage scenario #1. However, in this case, the 15 values of the monitoring feature identified from 15 tests were dispersed outside the confident region which could be due to the complex property change in the monitored structure. Although the feature values were distributed in a rather random manner, all of these values were outside this range, which manifested the need for further data downloading and analysis. It is seen in Figure 7e that the reduction in the mean values of K3, K4 and K5 when compared with those of the original structure verified the effectiveness of the system ID-based damage detection method.

#### 4.3.3. Damage Scenario #3

**Figure 8:**
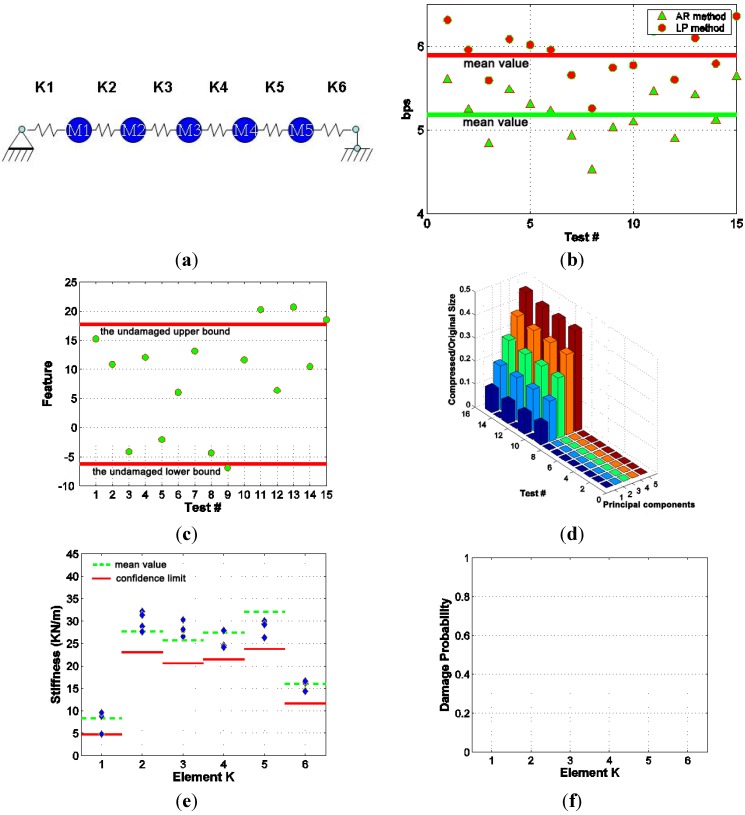
Performance of Integrated SHM system in Scenario #3 (**a**) Retrofitted structure (w/o damage); (**b**) Sensor data compression; (**c**) Feature monitoring; (**d**) Progressive data transmission; (**e**) Identified stiffness; (**f**) Statistical analysis of damage.

In this case, all disattached steel stripes were connected back to the primary structure by tightening the bolts at corresponding connections. In this way, a scenario which simulates retrofitted structure is created. The performance of the integrated SHM system in this scenario is presented in Figure 8. The compression performance of both the LP and AR methods was improved compared with the previous two cases with damage introduced to the system. The final bit rates of these two compression methods in this case were almost the same as those of the pre-test data. The monitoring feature suggests that most of the test data can be confidently classified as undamaged and only four test measurements (corresponding to test #9, #11, #13 and #15) indicate the necessity to be downloaded and further analyzed from observation of the feature. This clearly demonstrates the benefits of using the interactive data retrieval method to alleviate the data transmission and data analysis burden. After observing the PCA feature, the 4 test measurements which were not confidently classified as undamaged case can be progressively downloaded and analyzed. The system identification results show that the structure was restored to its original state and no stiffness reduction was observed.

## 5. Conclusions

This paper presents a new concept for an integrated structural health monitoring (SHM) system, which has a few desirable functionalities, including embedded sensor data compression, interactive sensor data retrieval, and structural knowledge discovery. The streamlined data flow in the integrated SHM system is used as a unifying thread to integrate the individual components of an on-line SHM system. Special consideration of its data flow aspect when designing and implementing an on-line SHM system will be very beneficial to enhancing its overall performance with features such as streamlined data flow and reduced load for data analysis. Adoption of this new concept will enable the design of more uniform data generation and data handling capacity in all the subsystems of an on-line SHM system. This is especially true for systems involving wireless sensor networks or wireless communication, which typically have a limited communication bandwidth.

To examine this concept in the context of vibration-based SHM system, real sensor data from an on-line SHM system comprising a scaled steel bridge and an on-line data acquisition system with remote data access was used in this study. Vibration tests conducted clearly demonstrated the prominent performance characteristics of such an integrated SHM system including rapid data access, interactive data retrieval and knowledge discovery of structural conditions on a global level. The results of this study confirm that implementing this concept in long-term on-line SHM systems is beneficial to enhancing the system’s operating efficiency and robustness, especially for SHM systems installed on civil infrastructures that typically have hundreds of sensors and may serve user groups at different geographical locations and with different knowledge needs.
